# Hospital at Home for Elderly COVID-19 Patients: A Preliminary Report with 100 Patients

**DOI:** 10.3390/jcm11071850

**Published:** 2022-03-26

**Authors:** Yuki Miyamoto, Tasuku Matsuyama, Katsutomo Kunimitsu, Hiroyuki Nagano, Yoshie Yamada, Shigemi Murakami, Yoshihiro Yamahata, Bon Ohta, Yoshiki Morikami, Masanori Nakagawa

**Affiliations:** 1 Department of Emergency and Disaster Medical Systems, Graduate School of Medicine, Kyoto Prefectural University of Medicine, Kyoto 6028566, Japan; task-m@koto.kpu-m.ac.jp (T.M.); k.kunimitsu@hcfm.jp (K.K.); yamahata@koto.kpu-m.ac.jp (Y.Y.); ohta2010@wind.ocn.ne.jp (B.O.); 2 Yoshiki Home Care Clinic, Kyoto 6158262, Japan; nagano.hiroyuki.55r@st.kyoto-u.ac.jp (H.N.); yamayosi89@gmail.com (Y.Y.); yoshiki.morikami@yoc.or.jp (Y.M.); 3 Department of Healthcare Economics and Quality Management, Graduate School of Medicine, Kyoto University, Kyoto 6068501, Japan; 4 Department of Healthcare Epidemiology, Graduate School of Medicine and Public Health, Kyoto University, Kyoto 6068501, Japan; 5 Watanabe Nishigamo Home Care Clinic, Kyoto 6038832, Japan; murakami@miyakokai.or.jp; 6 Department of Health and Welfare, Kyoto Prefectural Government, Kyoto 6028570, Japan; m-nakagawa96@pref.kyoto.lg.jp

**Keywords:** COVID-19, hospital at home, elderly, patient surge, healthcare burden, home care medicine, home death, cardiac arrest

## Abstract

Hospital-at-home (HaH) care is useful for patients with COVID-19 and an alternative strategy when hospital capacity is under pressure due to patient surges. However, the efficacy and safety of HaH in elderly patients with COVID-19 remain unknown. In Kyoto city, we conducted a retrospective medical record review of HaH care focused on elderly COVID-19 patients from 4 February to 25 June 2021. Eligible patients were (1) COVID-19 patients aged ≥70 years and those who lived with them or (2) COVID-19 patients aged <70 years with special circumstances and those who lived with them. During the study period, 100 patients received HaH care. Their median age was 76 years (interquartile range 56–83), and 65% were over 70 years. Among 100 patients, 36 (36%) had hypoxia (oxygen saturation ≤ 92%), 21 (21%) received steroid medication, and 34 (34%) received intravenous fluids. Although 22 patients were admitted to the hospital and 3 patients died there, no patients died during HaH care. HaH care may be safe and effective in elderly patients with COVID-19. Our study shows that HaH provides an alternative strategy for treating COVID-19 patients and can reduce the healthcare burden at hospitals.

## 1. Introduction

The coronavirus disease (COVID-19) pandemic has imposed unprecedented pressure on hospitals and other healthcare resources [1,2,3]. Customarily, patients with hypoxia, severe dehydration due to low oral intake, and risk factors for progression to severe conditions, including advanced age are hospitalized [4,5,6]. However, due to the COVID-19 surge, in many countries, including Japan, these patients are forced to stay at home and await hospitalization [7,8,9], thereby remaining in danger, and the absence of appropriate medical care may lead to patient death [9].

Hospital-at-home (HaH) care is an alternative strategy to inpatient care that provides acute hospital-level care within the patients’ homes to enable admission avoidance and early discharge [10,11,12,13,14]. HaH is an effective and safe healthcare model even for older patients with various diseases, including bacterial pneumonia, influenza, and heart failure [10,11,12,13,14]. Therefore, HaH may be useful when hospital capacity is particularly overextended due to COVID-19 [15]. However, only a few studies have investigated the effect of HaH care in COVID-19 patients, and none of these studies has focused on older patients [16,17].

In March 2020, the Kyoto Prefecture established the Kyoto Prefectural Admission Control Center for COVID-19 patients to coordinate admission and collect information from local public health centers for all COVID-19 patients in the prefecture. Based on the patients’ clinical severity and situation, doctors in the Admission Control Center would determine the patient’s disposition, including (1) hospital admission; (2) isolation at hotels, as quarantine accommodations; (3) staying at home and monitoring, via telephone calls from the local public health center. However, in the quarantine accommodations, patients need to be able to care for themselves, and there was an inadequate number of medical staff to provide physical care [9]. Thus, many older patients, especially those who need nursing care, could not stay at the quarantine accommodations.

When the number of beds was insufficient, critically ill patients were prioritized for hospitalization. Thus, older patients who could not care for themselves and had mild or moderate illness had no choice but to wait at home and be monitored by telephone calls. However, the local health center could not determine via telephone calls whether the condition of these older patients had deteriorated, if the condition was not fully explained by the patient, or the condition could not be diagnosed by the local health center staff; these problems could lead to the patients’ death without the provision of appropriate medical care.

To address these problems, the Kyoto Prefecture administration organized a special team for HaH care. Then, in Kyoto City, Japan, the administrative HaH care service commenced for older COVID-19 patients and for those who lived with the older patients from 4 February 2021. This HaH care system included an initial in-person physician visit followed by medical and nursing care provided through an in-person multidisciplinary service.

In this study, we describe the characteristics and outcomes of patients who received HaH care to evaluate the HaH care strategy for older patients.

## 2. Materials and Methods

### 2.1. Study Design and Setting

We conducted a retrospective medical record review of older COVID-19 patients who received HaH care in Kyoto City, the prefectural capital of Kyoto. The Population Census of 2020 revealed that the total population of Kyoto Prefecture and Kyoto City were 2.58 and 1.46 million, respectively [18]. On 4 February 2021, Kyoto City had 6123 COVID-19 patients. In Kyoto Prefecture, the reserved bed capacity for COVID-19 patients was 416, including 86 beds for critically ill patients [19].

When the Admission Control Center selects a patient as eligible for HaH care, the HaH team is provided with detailed information about the patient and starts treatment (Figure S1 in Supplemental Materials). The HaH care multidisciplinary team comprises doctors, nurses, pharmacists, rehabilitation therapists, care workers, medical oxygen suppliers, and assistive product providers and provides hospital-level full-time care, including daily physician and nurse visits, and telephonic examinations. Older patients were mandatorily treated face-to-face at least once a day. Patients with high medical needs or those with high risks (e.g., rapid clinical deterioration, the requirement of intravenous fluids, or living alone with dementia) were visited by a physician once a day and by a nurse once or twice a day. However, the frequency of visits was sometimes reduced to once every 2 days, along with a telephonic medical check-up for patients with very mild illness. At night, in principle, doctors visited and treated patients within 1 h of the patient’s call. HaH physicians and nurses could perform blood tests, cultures, electrocardiograms, ultrasounds, oral drug administration, intravenous drug administration, intravenous fluid administration, and oxygen administration according to the prescribed protocol (Figures S2 and S3 in the Supplemental Materials). Blood tests were obligatorily performed at the first visit to detect any unidentified underlying diseases (e.g., diabetes, liver, or kidney disease). Thereafter, these tests and treatments were performed according to the patient’s condition. For patients with hypoxia, oxygen and steroid medications were administered. Notably, physicians could not administer antiviral drugs (e.g., favipiravir or remdesivir) in HaH care because the Ministry of Health, Labor, and Welfare had approved the administration of such antiviral drugs only for use in the hospital setting at that time. If physicians required further examinations (e.g., computed tomography scanning), patients were transferred to the hospital as outpatients by special vehicles designated for COVID-19 patients. In case of worsening clinical status, the patient was referred to the Admission Control Center by the HaH physicians and subsequently transferred to the hospital by ambulance.

### 2.2. Definition of COVID-19 and Eligibility Criteria for HaH Patients

We included consecutively treated COVID-19 patients enrolled from 4 February to 25 June 2021. The diagnosis of COVID-19 was based on nucleic acid amplification tests or antigen tests. The eligibility criteria for receiving HaH care were (1) COVID-19 patients aged ≥70 years and patients who lived with these older patients or (2) COVID-19 patients younger than 70 years with special circumstances (e.g., childcare, elder care, mental illness, or limited proficiency in Japanese) and those who lived with these patients. Patients who had severe respiratory distress (e.g., requiring immediate tracheal intubation or non-invasive positive pressure ventilation therapy) were excluded from this analysis.

### 2.3. Ethics

The Institutional Review Board of Yoshiki Home Care Clinic approved the study protocol. Informed consent was obtained from all patients who received HaH care. As obtaining written consent posed a risk for contagious infection, all consents were obtained orally, and the physicians recorded the consent status on the consent form.

### 2.4. Data Collection and Statistical Analysis

We collected data from the electronic health records to describe COVID-19 patients who received HaH care. Patient characteristics included age and sex. Furthermore, we included details such as the intervention period, number of physician visits, number of nurse visits, number of pharmacist visits, patients’ symptoms during the intervention period, medical treatment, and patient outcome. In addition, we described the characteristics, intervention, and outcome of patients aged ≥70 years. The characteristics of older patients included their score on the Clinical Frailty Scale (CFS) 2 weeks prior to commencement of HaH care [20,21,22]. Continuous variables are presented as the median and interquartile range (IQR), whereas categorical data are expressed as numbers (%). Analyses were performed using Stata/MP version 16 (StataCorp, College Station, TX, USA).

## 3. Results

During the study period, 5293 patients were diagnosed with COVID-19 in Kyoto City. Of these, 100 patients (1.9%) received HaH care. Table 1 shows the baseline characteristics, symptoms, and clinical outcomes of all patients who received HaH care. The median age was 76 (IQR 56–83) years, and 57% were female. The median time from onset to intervention was 4 days (IQR 2–7), and the median time from diagnosis to intervention was 1 day (IQR 1–3). The median intervention period was 10 days (IQR 7–14). During the intervention period, the median number of visits by doctors, nurses, and pharmacists, as well as telephonic examinations, were 6.5 (IQR 4–9), 5.5 (IQR 0–12), 1 (IQR 0–2), and 2 (IQR 1–4), respectively.

With regard to patient symptoms, 75% of patients had a fever of ≥37.3 °C, 54% had a fever of ≥38.0 °C, and 36% had hypoxia (oxygen saturation ≤ 92%) during the intervention period. Approximately one-third of the patients received oxygen therapy, and steroid medication was administered to 21% of patients. Moreover, two patients received non-invasive positive pressure ventilation at their homes. In total, 22 patients (22%) were referred to the control center and hospitalized, and 3 of the hospitalized patients died there (age, 75, 80, and 90 years; time from admission to death 16, 16, and 24 days, respectively). No patient died during HaH care. Among the patients who had hypoxia, 56% (20/36) recovered without hospitalization.

The baseline characteristics, symptoms, and clinical outcomes of patients aged 70 years or older are shown in Table 2. There were 65 patients aged ≥70 years and their median age was 81 (IQR 76–87) years, and the median CFS was 4 (IQR 3–6). During the intervention period, patients with a fever of ≥37.3 °C, a fever of ≥38.0 °C, and with hypoxia accounted for approximately 80%, 58%, and 45% of the study population, respectively. Supplementary oxygen therapy or steroids were administered to 38% and 24% of patients, respectively, and 72% (47/65) of COVID-19 patients aged 70 years or older recovered without hospitalization. Among patients who had hypoxia and were aged 70 years or more, 48% of patients (14/29) recovered with HaH care alone.

## 4. Discussion

In this study, we retrospectively described the characteristics, treatment, and outcomes of HaH patients with COVID-19, and 78% of COVID-19 patients who received HaH care recovered with only HaH care; no patient died in their home during HaH care. Moreover, our study showed that nearly half of the patients who had hypoxia recovered from COVID-19 with HaH care alone despite being 70 years of age or older. Given the results of our study, we believe that HaH care for older COVID-19 patients could be safe and effective for admission avoidance and that HaH care could have spared the number of hospital beds, suggesting the possibility that HaH may contribute to alleviating the shortage of hospital beds.

One of the strengths of our study is that HaH care mainly focused on older patients. Several studies have reported on HaH care for COVID-19 patients; however, in these studies, high-risk patients were excluded, including those aged ≥65 years. Thus, the previous studies focused on young and middle-aged COVID-19 patients (median age early 50s) [16,17]. In comparison, the median age of the patients in our study was 76 years. Another strength of this study is that the clinical severity of the participants was higher than those in previous studies. For example, the proportion of patients who received oxygen therapy was 32% in our HaH care, compared with 22% in the largest earlier study [17]. Similarly, 34% of patients in our study received intravenous fluids, whereas only 11% of patients received intravenous therapy in a previous study [17]. These differences in severity may be attributable to the advanced age and frailty of our patients. Furthermore, we investigated the CFS score of older patients. Among the patients aged ≥70 years, nearly half had a CFS score of 5–8. A high CFS score has been associated with delirium, falls, worsening of cognitive function, and prolonged hospitalization [23]. Thus, we believe that offering the option of HaH to older COVID-19 patients with high CFS scores could be of great benefit to both patients and healthcare providers.

Regarding the rate of transfer to the hospital during HaH interventions, a large case–control study, which mainly focused on elderly patients with various acute medical illnesses, showed that 12% of HaH patients were transferred to the hospital [11]. Similarly, retrospective studies in patients with COVID-19 described that 13% of patients were transferred to inpatient hospitalization [17]. In our study, 23% of HaH patients were hospitalized, slightly higher than these previous studies. This difference in hospitalization between COVID-19 and other acute medical illnesses may be because there was no definitive therapy in patients with COVID-19, which is exemplified by antibiotics for bacterial infection. In addition, the difference in hospitalization rate between our study and previous studies of COVID-19 HaH care could be due to patient characteristics, including age and severity of diseases. On the other hand, the mortality of the patients who received HaH care in our study was 3%, which was compatible with previous HaH studies that focused on elderly patients [24]. Considering these results, we believe the HaH care model described in this study appears to be safe and effective in view of the clinical outcomes. We recognize that the key to the success of COVID-19 HaH care includes two factors: (1) patient care and (2) medical systems. With regard to patient care, we believe that frequent visits and observations by doctors and nurses should be essential. A previous study suggested that receiving telemedicine service constitutes a great challenge for older patients because of difficulties in hearing and communicating, as well as dementia [25]. In addition, COVID-19 patients have another problem—the so-called “silent hypoxia” or “happy hypoxia”—in which oxygen saturation is reduced without any signs of respiratory distress [26,27,28]. Thus, disease progression can be easily overlooked, particularly in older COVID-19 patients who are at high risk of deterioration when examined only telephonically. Therefore, we believe that daily face-to-face examination is key to preventing home death in patients with COVID-19. With regard to the medical system, it is important that the system allows physicians to promptly consult with the Admission Control Center in case of worsening of the patient’s clinical condition. As described above, in the Kyoto Prefecture, the Admission Control Center collects information for all COVID-19 patients and decides their disposition. Before we began the HaH care system, the local public health center telephonically followed up on the condition of patients who were forced to stay at home and, when these patients experienced clinical worsening, they would be referred to the Admission Control Center. Inaccurate triage resulting from a lack of face-to-face assessment may overlook COVID-19 patients, particularly older patients, who in fact need hospitalization. The HaH care service provides more accurate information to the Admission Control Center based on the quick detection of the patient’s worsening condition.

Several limitations of this study should be acknowledged. First, the reproducibility of the results is limited because the criteria for providing HaH care and that of hospitalization from HaH varied depending on the number of hospitalized patients. Second, as medical systems and the prevalence of COVID-19 vary by country and region, the external validity is limited. Third, because of the descriptive study design, we could not investigate the actual causal relationship between HaH care and the safety or efficacy of care in older COVID-19 patients. Further large-scale studies, especially randomized clinical trials, are required to verify the findings of this research.

In conclusion, HaH care can be safe and effective for COVID-19 patients, even in those at an advanced age, thereby providing an alternative care strategy for COVID-19 patients and reducing the healthcare burden in hospitals.

## Figures and Tables

**Table 1:** Baseline characteristics and outcomes of study population (*n* = 100).

**Characteristics**	
Age, years, median (IQR)	76 (56–83)
Male, *n* (%)	43 (43)
Time from onset to intervention, days, median (IQR)	4 (2–7)
Time from diagnosis to intervention, days, median (IQR)	1 (1–3.25)
Intervention period, days, median (IQR)	10 (7–14)
Number of visits	
Doctor, *n*, median (IQR)	6.5 (4–9)
Nurse, *n*, median (IQR)	5.5 (0–12)
Pharmacist, *n*, median (IQR)	1 (0–2)
Number of telephone examination, *n*, median (IQR)	2 (1–4)
Patients’ symptoms	
Fever (≥37.3 °C), *n* (%)	75 (75)
Fever (≥38.0 °C), *n* (%)	54 (54)
Hypoxia (SpO2 ≤ 92%), *n* (%)	36 (36)
Intervention	
Frequency of blood test, *n*, median (IQR)	2 (1–4)
Intravenous fluid administration, *n* (%)	34 (34)
Oxygen administration, *n* (%)	32 (32)
Steroid administration, *n* (%)	21 (21)
Ultrasound (lung, heart, lower extremity), *n* (%)	9 (9)
**Outcomes**	
Hospitalized, *n* (%)	22 (22)
In-hospital death, *n* (%)	3 (3)

IQR, interquartile range; SpO2, oxygen saturation.

**Table 2:** Baseline characteristics and outcomes of patients aged ≥70 years (*n* = 65).

**Characteristics**	
Age, years, median (IQR)	81 (76–87)
Male, *n* (%)	26 (40)
Clinical frailty scale 2 weeks prior to first visit, median (IQR)	4 (3–6)
Time from onset to intervention, days, median (IQR)	4 (2–7)
Time from diagnosis to intervention, days, median (IQR)	1 (1–3)
Intervention period, days, median (IQR)	10 (7–15)
Number of visits	
Doctor, *n*, median (IQR)	7 (4–9)
Nurse, *n*, median (IQR)	7 (0–14)
Pharmacist, *n*, median (IQR)	1 (0–3)
Number of telephone examination, *n*, median (IQR)	2 (1–5)
Patients’ symptom	
Fever (≥37.3 °C), *n* (%)	52 (80)
Fever (≥38.0 °C), *n* (%)	38 (58)
Hypoxia (SpO2 ≤ 92%), *n* (%)	29 (45)
Intervention	
Frequency of blood test, *n*, median (IQR)	2 (1–4)
Intravenous fluid administration, *n* (%)	30 (46)
Oxygen administration, *n* (%)	25 (38)
Steroid administration, *n* (%)	16 (24)
Ultrasound (lung, heart, lower extremity), *n* (%)	6 (9.2)
**Outcomes**	
Hospitalized, *n* (%)	15 (23)
In-hospital death, *n* (%)	3 (4.6)

## Data Availability

The data presented in this study are available on request from the corresponding author. The data are not publicly available due to patients’ privacy.

## Supplementary Materials

The following supporting information can be downloaded at: https://www.mdpi.com/article/10.3390/jcm11071850/s1, Figure S1: Flowchart of hospital-at-home care to eligible patients, Figure S2: Clinical protocol for hospital-at-home care in Kyoto City, Figure S3: Examination and treatment protocol of hospital-at-home care at each visit.
