# Supplementary material for: Hospital at Home for Elderly COVID-19 Patients: A Preliminary Report with 100 Patients
Source: J Clin Med. 2022 Mar 26;11(7):1850. doi: 10.3390/jcm11071850 (PMC8999675; doi:10.3390/jcm11071850)
Supplement: Supplementary file 1 [file jcm-11-01850-s001.zip › jcm-1623543-supplementary.pdf]

Legends of Supplemental Materials

Figure S1. Flowchart of Hospital-at-Home care to eligible patients

Figure S2. Clinical protocol for Hospital-at-Home care in Kyoto City

Figure S3. Examination and Treatment Protocol of Hospital-at-Home Care at Each Visit

Figure S1. Flowchart of providing hospital-at-Home care to eligible patients

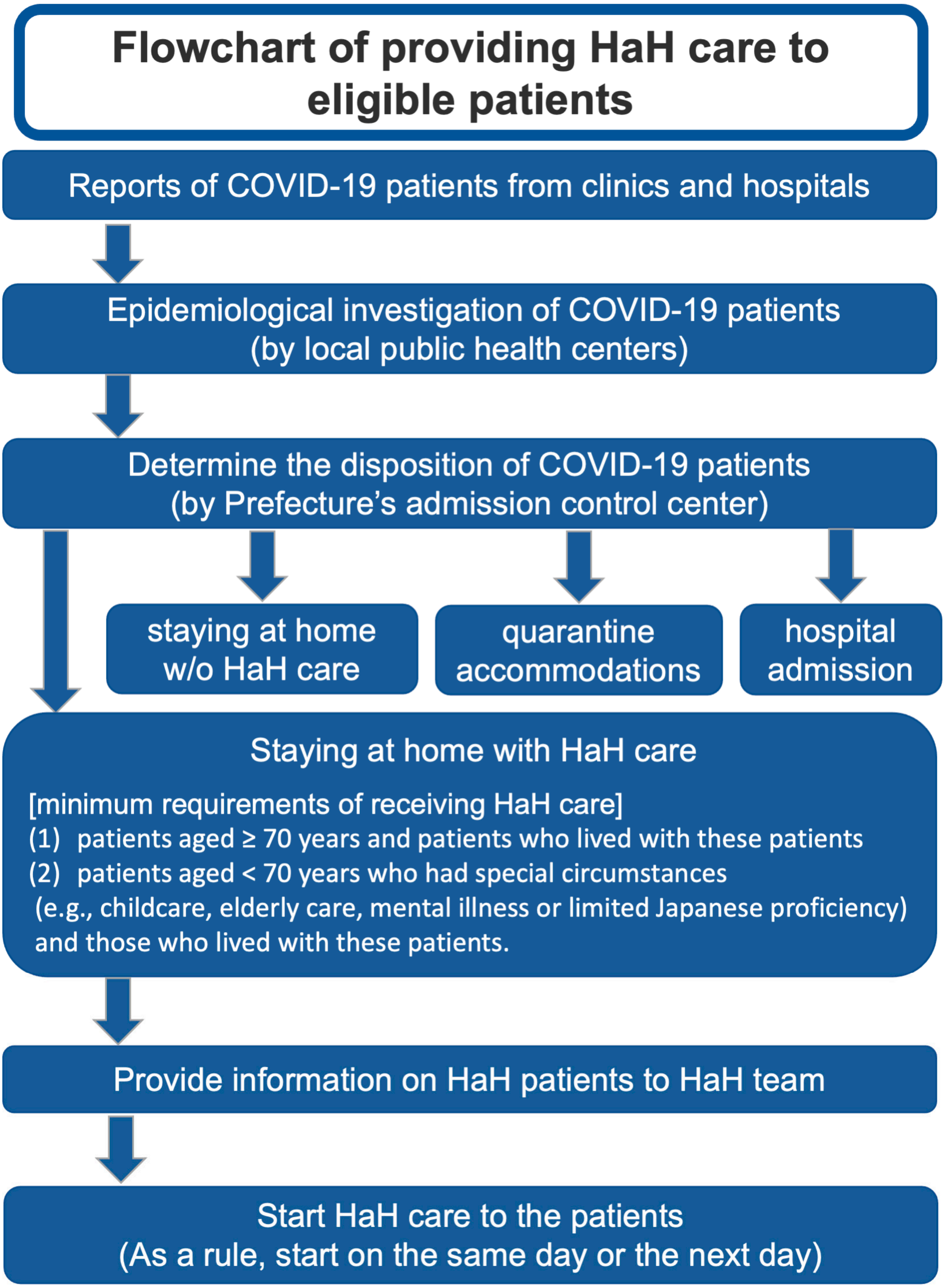

Note that this flowchart was used during the study period and may have changed subsequently after the study period.

Figure S2. Clinical Protocol for Hospital-at-Home care in Kyoto City

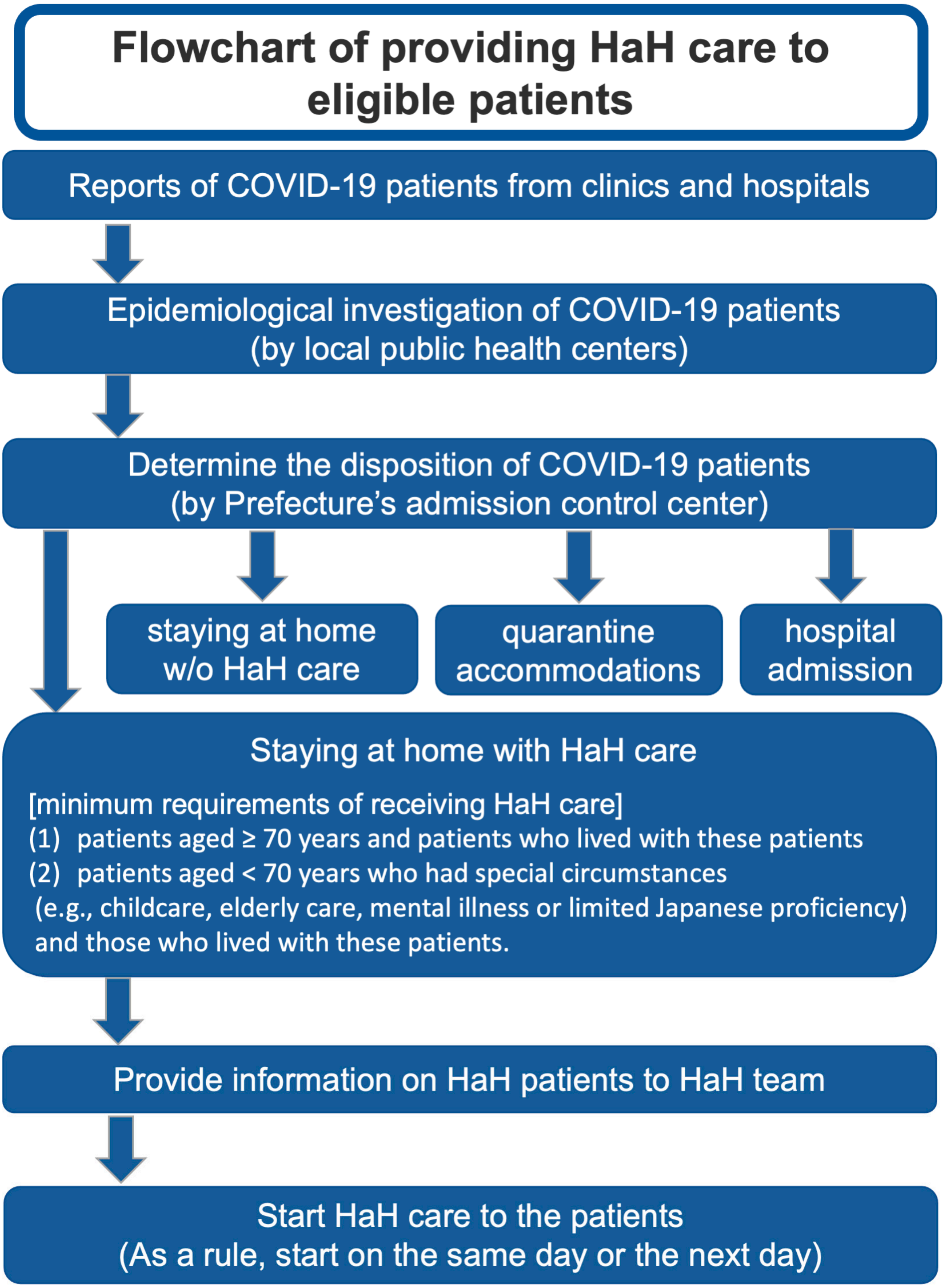

Note that this flowchart was used during the study period and may have changed subsequently after the study period.

Figure S3. Examination and treatment protocol of hospital-at-home care at each visit

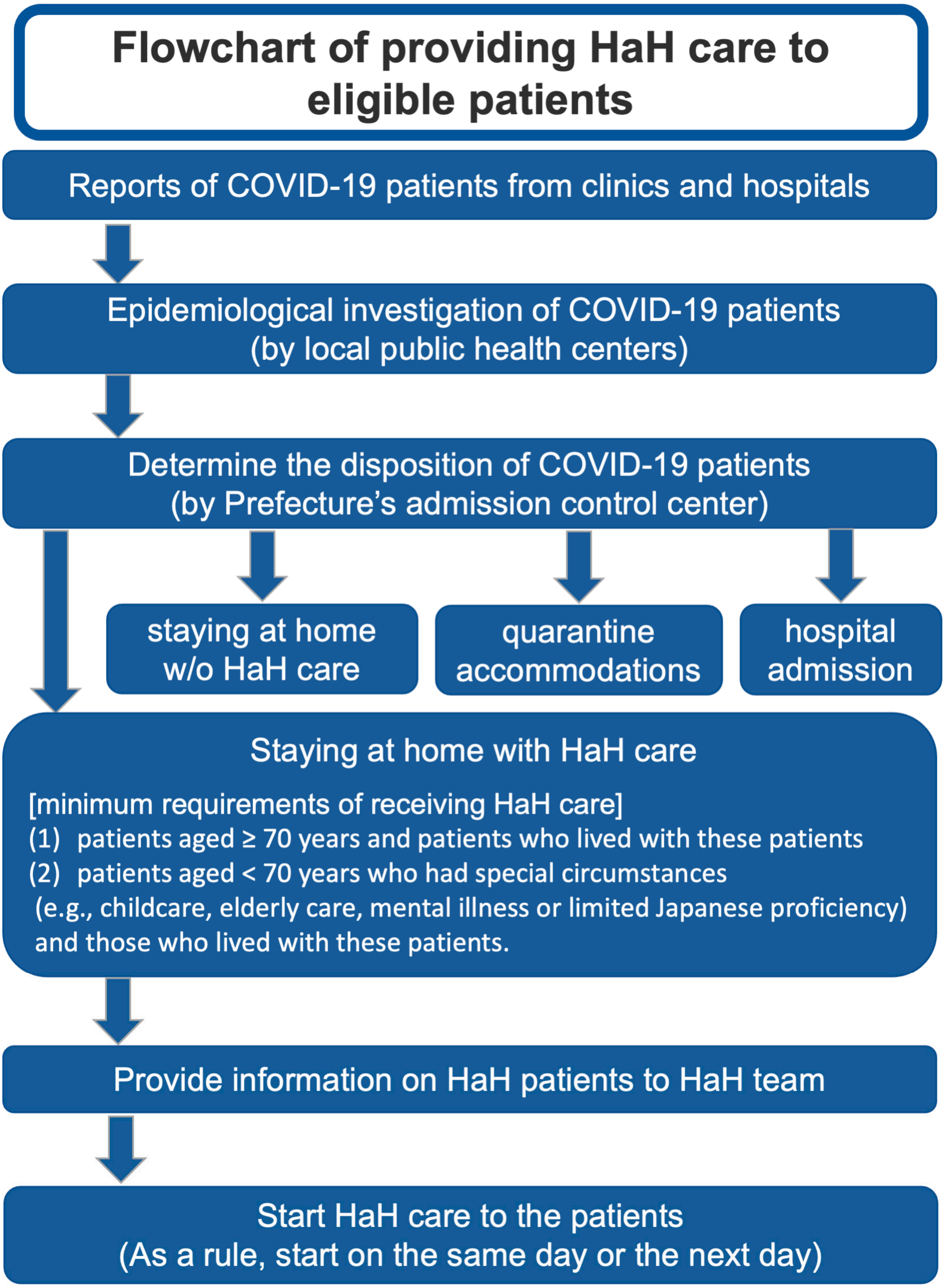

Note that this flowchart was used during the study period and may have changed subsequently after study period.
